# Post-activation performance enhancement of flywheel training on lower limb explosive power performance

**DOI:** 10.3389/fphys.2023.1217045

**Published:** 2023-07-18

**Authors:** Keqi Fu, Lingying Chen, Eric Tsz-Chun Poon, Rou Wang, Qian Li, Haochong Liu, Indy Man Kit Ho

**Affiliations:** ^1^ Department of Public Physical, Zhejiang Chinese Medical University, Zhejiang, China; ^2^ Department of Health and Physical Education, The Education University of Hong Kong, Tai Po, Hong Kong, China; ^3^ Sports Coaching College, Beijing Sport University, Beijing, China; ^4^ School of Nursing and Health Studies, Hong Kong Metropolitan University, Hong Kong, China; ^5^ Asian Academy for Sports and Fitness Professionals, Hong Kong, China

**Keywords:** flywheel training, accentuated eccentric loading, warm up, jump, sprint

## Abstract

The study aimed to investigate the post-activation performance enhancement (PAPE) of flywheel training (FT) on lower limb explosive power performance. Using a randomized crossover design, 20 trained men (age = 21.5 ± 1.4 years; training experience 5.5 ± 1.2 years) completed seven main conditions after three familiarization sessions. The first three conditions tested the PAPE of the FT on the counter movement jump (CMJ) under three different inertial loads (0.041 kg·m^2^ as L; 0.057 kg·m^2^ as ML; and 0.122 kg·m^2^ as P), whereas the following four conditions tested the PAPE of FT on the 30 m sprint, which consisted of three inertial loads (L, ML, and P) and a control condition. Participants were required to perform the CMJ or 30 m sprint at baseline (Tb) and immediately (T0), 4 min (T4), 8 min (T8), 12 min (T12), and 16 min (T16) after exercise, respectively. The results of the CMJ conditions showed that PAPE peaked at T4 (*p* < 0.01) and almost subsided at T12 (*p* > 0.05) in ML and P conditions. Meanwhile, PAPE appeared earlier in the P condition, and the effect was more significant (P:ES = 1.09; ML:ES = 0.79). 30 m sprint results showed significant improvement only in the ML condition. The PAPE peaked at T4 (*p* < 0.05, ES = −0.47) and almost subsided at T8 (*p* > 0.05). It was mainly due to the significant enhancement of the 10–30 m segmental timing performance at T4 (*p* < 0.05, ES = −0.49). This study indicates that the size of the inertial load could influence the magnitude of the PAPE produced by the explosive force of the lower limb. The PAPE of the vertical explosive force increased with increasing inertial load, but the PAPE of the horizontal explosive force did not appear at the maximum inertial load. The most effective elicitation of the PAPE was at 4–8 min after the FT.

## 1 Introduction

Strength and power characteristics are highly demanded in many sports and conditioning drills (Philpott et al., 2021). Athletes are required to generate high levels of force and power in performing different movements and skills in a speedy manner such as acceleration, sprinting, jumping, stopping, and turning. In this regard, high strength and power output have been identified as the most important contributor to the success of various sports (Hori et al., 2009). To effectively monitor the individual power output in both the lab and field setting, vertical jump tests, such as counter movement jump (CMJ), and sprint test (e.g., 30 m sprint) are widely adopted (Souza et al., 2020). The quick and easy administration of these tests allows coaches to monitor the neuromuscular status and training progression without interfering with their training (Claudino et al., 2017). Meanwhile, strength coaches and sports scientists are always interested in exploring new exercise methods in acute enhancing jumping and sprinting performance.

Post-Activation Performance Enhancement (PAPE) (Cuenca-Fernandez et al., 2017) has increasingly become a topic of interest in sports science and exercise physiology, owing to its potential to improve athletic performance. PAPE was suggested to indicate the enhancement of maximal voluntary (dynamic or isometric) strength, power, or speed following a conditioning contraction. These enhancements of maximal and powerful performances are typically represented by improved strength or jumping and sprinting exercises (Blazevich and Babault, 2019; Prieske et al., 2020). This neuromuscular phenomenon refers to enhancing voluntary muscle performance following a conditioning activity, generally over a time window exceeding 5 min (Cuenca-Fernández et al., 2017; Cuenca-Fernández et al., 2019; Cuenca-Fernández et al., 2020; Cuenca-Fernández et al., 2023). Above all, it contributes significantly to understanding this mechanism and its practical applications in athletic performance enhancement. In light of these findings, the potential benefits of incorporating PAPE into warm-up before training and competition strategies become apparent, and the importance of further research into understanding its underlying mechanisms and optimal application is underscored.

Recently, flywheel training (FT) has emerged as an efficacious strategy for a wide range of athletes to enhance sports performance (Buonsenso et al., 2023). For instance, the physical capacities of soccer players (i.e., strength, power, jump, and direction changes) (Allen et al., 2021), running economy of distance runners (Weng et al., 2022), lower-body strength and power qualities in male academy rugby players (Murton et al., 2021), and the CMJ performance of basketball players (Stojanović et al., 2021) were shown to be significantly improved using FT. Strength and conditioning practitioners may acutely or chronically improve athletes’ performances with the FT as the eccentric phase is associated with a high mechanical overload (Gonzalo-Skok et al., 2017). It is also believed that the higher force output potential during the eccentric phase may maximize the stretch-shortening cycle (SSC) and force production in the subsequent concentric phase. The enhanced SSC performance can be further transferred to many explosive athletic tasks like vertical jumps, horizontal jumps, and sprinting (Beato et al., 2019).

Furthermore, it was proposed that a higher eccentric load may be positively associated with a greater PAPE by recruiting fast-twitch muscle fibers more effectively in sport-specific performance (Beato et al., 2019; Beato et al., 2021a). On the other hand, a recent study compared the PAPE difference between squat and deadlift FT exercises on isokinetic quadriceps and hamstrings. Despite the higher observable concentric power in FT-squat, no significant PAPE difference in hamstring and quadriceps isokinetic performance was found (Beato et al., 2020). Therefore, PAPE responses on these lower limb FT exercises with fundamental biomechanical difference seem comparable. The recent review conducted by Beato et al. (Beato et al., 2019) has given a provisional summary regarding the effective inertia intensities (0.03–0.11 kg·m^2^), volume (3 sets of a large force and power outputs exercises), and rest period (3–9 min).

Despite the extensive evidence on the use of FT and PAPE in jumping and sprinting performance enhancement, many studies in this review either did not clearly report the flywheel intensity or analyze the manipulation of inertial loads for performance, limiting the generalization of the result findings. In addition, only very few studies compared the difference between FT and traditional resistance modality. Many aspects regarding the FT-based PAPE are still not well understood or inconclusive. For example, the influence of prior traditional weightlifting experience on the FT-induced PAPE, and whether FT is more beneficial than traditional gravitational-based resistance methods are both unclear (Seitz and Haff, 2016; Bauer et al., 2019). Moreover, only very few studies addressed the change in the sprint performance (5–20 m), and these studies did not reveal all the essential training parameters (e.g., inertial load) explicitly (Cuenca-Fernández et al., 2015). In the recent study conducted by Beato et al. (Beato et al., 2021b), their FT condition using high load (0.061 kg·m^2^) seemed to be inducing a slightly more performance increase (although not reached statistical significance) in both CMJ height and peak power, and the change of direction 6 min after FT implemented. Theoretically, the use of higher FT volume or inertial load should increase the mechanical power output leading to higher metabolic demand and hence the potential increase of muscle temperature, whereas the muscle temperature was identified to be one of the most impactful positive factors for PAPE (Blazevich and Babault, 2019). However, most of the previous FT studies did not investigate and compare the magnitude of PAPE using the inertia over 0.1 kg·m^2^. It is therefore hypothesized that FT using different inertial loads (especially the condition over 0.1 kg·m^2^) could provide different PAPE responses on jumping and sprinting tasks.

To bridge some of the aforementioned research gaps, this study aimed to compare different inertial loads that could potentially provide different PAPE responses. We also aimed to outline the optimum FT-based PAPE when compared with the traditional resistance modality. Furthermore, several previous studies have only given at least 48 h of inter-session recovery (Beato et al., 2020; de Keijzer et al., 2020). Since muscle damage and soreness after performing unaccustomed or eccentric FT strength training can be highly prominent and reach the peak, especially for males in 48 h (Fernandez-Gonzalo et al., 2014), the complete elimination of post-session carryover fatigue using standard recovery interval (i.e. 48 h) is questionable. Therefore, a longer separation period (i.e., at least 72 h) was adopted to minimize the uncertain impact in this regard. By comparing the FT and gravitational-based strength exercise using a wide spectrum of inertial loads and post-activation rest intervals the acute effect on sport-specific, lower limb explosive power performance (CMJ and sprint) can be better informed.

## 2 Materials and methods

### 2.1 Participants

Twenty healthy trained men were recruited into the study (age 21.5 ± 1.4 years, height 177.5 ± 5.2 cm, weight 74.6 ± 5.8 kg, training experience 5.5 ± 1.2 years). Participants in this study must meet the following inclusion criteria to minimize potential biases: 1) free from lower extremity injuries in the past 3 months and; 2) with a minimum of 3 years of strength training experience at least 3 days per week; 3) could squat at least 1.5 times of their body weight. All procedures conformed to the Declaration of Helsinki. Informed consent were acquired prior to the experiment with all the benefits and potential risks associated with the study explained to participants.

### 2.2 Experimental procedures

**FIGURE 1:**
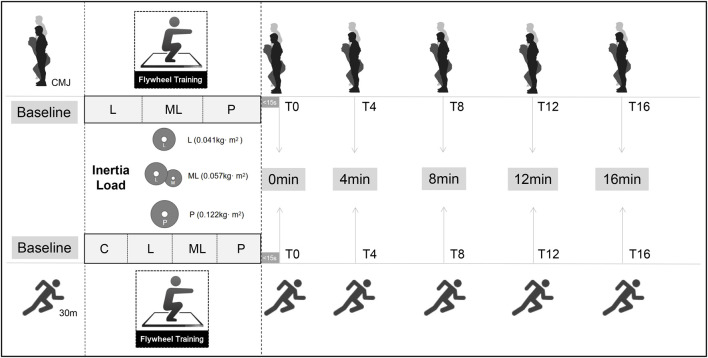
Experimental procedure diagram.

The study was conducted in a randomized crossover design, with each subject required to complete a total of seven main trials (three CMJ conditions and four sprint conditions). All trials were performed at least 72 h apart to eliminate fatigue or carryover effects (Figure 1). All participants completed three familiarization sessions of FT to fully understand and get used to the proper FT techniques before the main trials. The baseline values of CMJ or 30 m sprint (depending on the experimental conditions) were acquired for comparison in each of the seven conditions. The three CMJ conditions were conducted to investigate the PAPE of the FT on the CMJ under different inertial loads (0.041 kg·m^2^ as large [L]; 0.057 kg·m^2^ as medium-large [ML]; and 0.122 kg·m^2^ as Pro [P]). Subjects performed the CMJ trials before, and immediately (within 15 s; T0), 4 min (T4), 8 min (T8), 12 min (T12), and 16 min (T16) after the flywheel intervention, respectively. Similarly, subjects completed four 30 m sprint conditions using L, ML, and P inertial loads of FT and a controlled condition without intervention using the same time point as the CMJ conditions. A standardized 15-minute warm-up protocol was used in each session before the baseline test, including a 10-minute cycling at constant power (1 W per kg of body mass) and a 5-minute dynamic warm-up drills focusing on the hip, knee, and ankle as well as mimicking the squat, jumping and sprinting movements.

#### 2.2.1 Flywheel intervention

The experimental intervention protocol consisted of 4 sets of 7 repetitions of half squat exercise (above parallel) using a flywheel device (DESMOTEC, Italy). After the completion of a 15-minute standardized warm-up protocol, participants were required to perform each repetition at maximal velocity interspersed by a 3-minute inter-set passive rest (Seitz and Haff, 2016; Beato et al., 2020). The squat kinematics and quality were monitored and immediately feedback by an investigator with extensive strength training experience. Standardized verbal encouragements were provided to safeguard the maximum movement speed of each repetition. Three specific inertial loads (L, ML, and P; described above) were adopted in different conditions. For each set of FT squats, two additional preparator repetitions with partial range and speed were given to facilitate the flywheel recoil and maximum loading speed in the subsequent seven training repetitions. During FT, relevant parameters (e.g., average velocity) were monitored based on the participants’ performance so that appropriate adjustments could be made.

#### 2.2.2 CMJ trials

CMJ trials were measured by a HD force platform (Hawkin Dynamics Inc., United States; 1,000 Hz) (Badby et al., 2022). Participants stepped on the force plates and stood completely upright (extended hips and knees) and motionless for at least one second before completing a maximum CMJ with arms akimbo after the command “3, 2, 1, begin” given. Participants were cued to jump “as high as possible” for three CMJ trials whereas the average value was used for further analyses. An excellent test-retest intraday reliability (ICC = 0.906) was observed while the smallest worthwhile change (SWC) was 0.53 cm.

#### 2.2.3 30 m sprint trials

The 30 m sprint trials were measured by the Smart Speed timing Gates System (Fusion Sport Inc., Australia) whereas the timing gates were placed at the starting point, 10 m and 30 m. Subjects stood at the starting point in a ready position and sprinted to the finish line at full speed after the “3, 2, 1, go” command was given. The test was conducted once and the split times were recorded. A good (ICC = 0.881) test-retest inter-day reliability was found and the SWC was 0.01 s and 0.02 s for 10 m and 30 m respectively.

### 2.3 Statistical analyses

The data were analyzed by SPSS 23.0 while descriptive statistics were presented using the mean ± standard deviation ( $\bar{x}$ ±s). The test-retest intraday and inter-day reliability (during the baseline measurement) was assessed using the intraclass correlation coefficient (ICC). The ICCs are classified as ≥ 0.9 = excellent; 0.9 ≥ ICC ≥0.8 = good; 0.8 ≥ ICC ≥0.7 = acceptable; 0.7 ≥ ICC ≥0.6 – questionable; 0.6 ≥ ICC ≥0.5 = poor; ICC ≤0.5 = unacceptable (Beato et al., 2019). Normality tests of dependent variables were verified and all passed with Skewness-Kurtosis tests. The change of PAPE within conditions was calculated by percentage differences (diff%) and the formula is as follows: (Ti–baseline)/baseline × 100, with i representing any time point of CMJ/30 m sprint trial after the intervention. The difference between conditions was compared using two-way repeated measures ANOVA (Time × Condition). The significance level was set at *p* < 0.05. Cohen’s d effect sizes (ES) were calculated from the original data to quantify the magnitude of the difference of PAPEs. The ES is classified as small = 0.2, moderate = 0.5, and large = 0.8 (Cohen, 1988).

## 3 Results

### 3.1 PAPE of different inertial loads on CMJ

**FIGURE 2:**
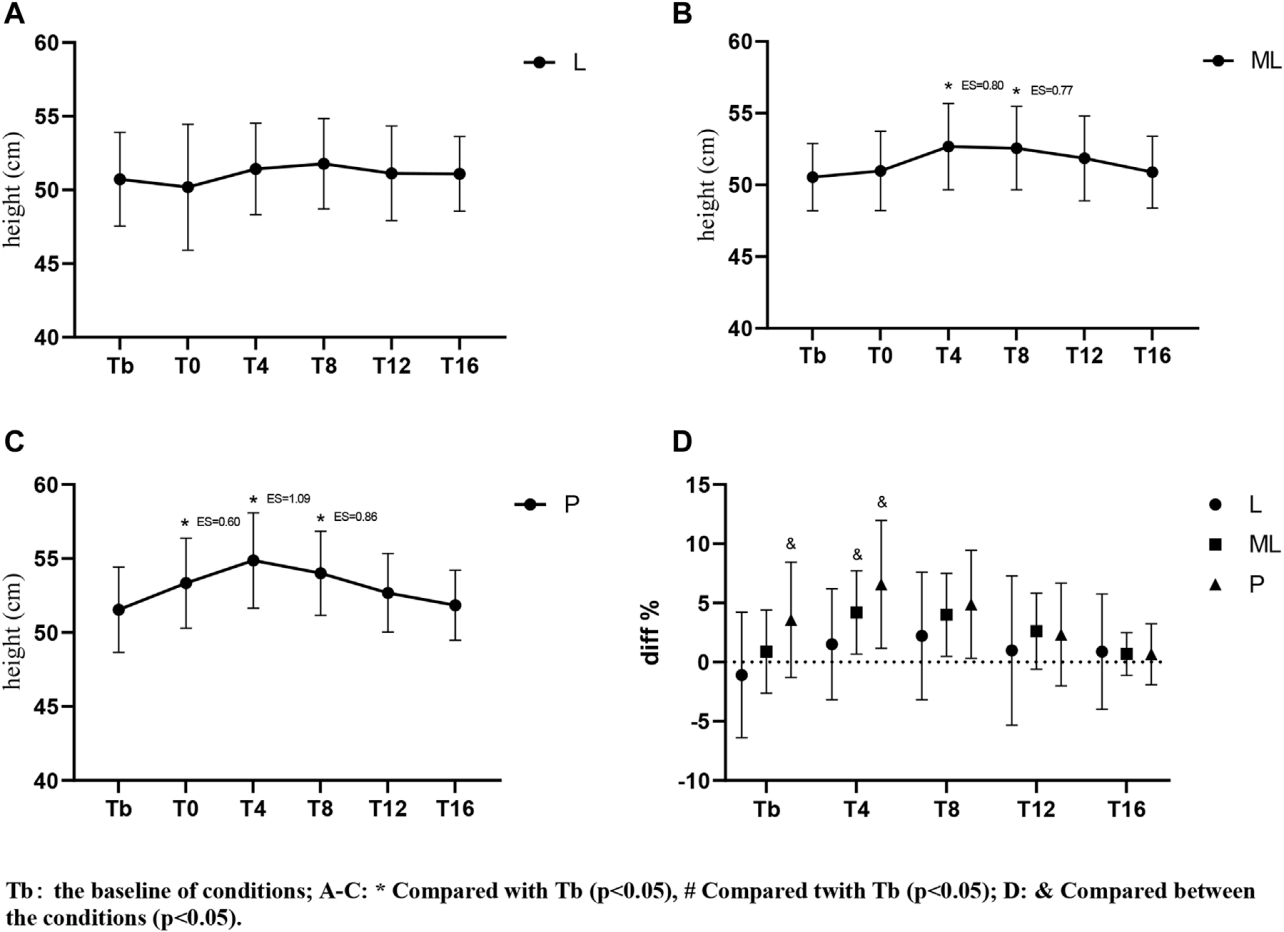
Variation of PAPE in CMJ with time under different inertial loads.

The two-way repeated ANOVA showed significant differences (F(4,80) = 5.008, *p* = 0.001) of condition and time interaction on PAPE. The simple effects of the CMJ showed a trend that PAPE peaked at T4 (*p* < 0.01) and almost subsided at T12 (*p* > 0.05) in ML and P conditions. Regarding the magnitude of effect, T4 showed large (ES = 1.09) and moderate (ES = 0.79) effects on P and ML conditions respectively when compared to the pre-test baseline (Tp). Meanwhile, an earlier significant PAPE was observed in the P condition (T0, *p* = 0.003; ES = 0.60) only but not in L or ML conditions (T0, *p* > 0.05). Conversely, there was no significant CMJ difference before and after the intervention in the L condition (Figure 2). When comparing the diff% between conditions, the ML condition showed a significant difference at T4 (*p* = 0.049) whereas both T0 (*p* = 0.003) and T4 (*p* = 0.005) showed significant differences in the P condition.

### 3.2 PAPE of different inertial loads on 30 m sprint

**FIGURE 3:**
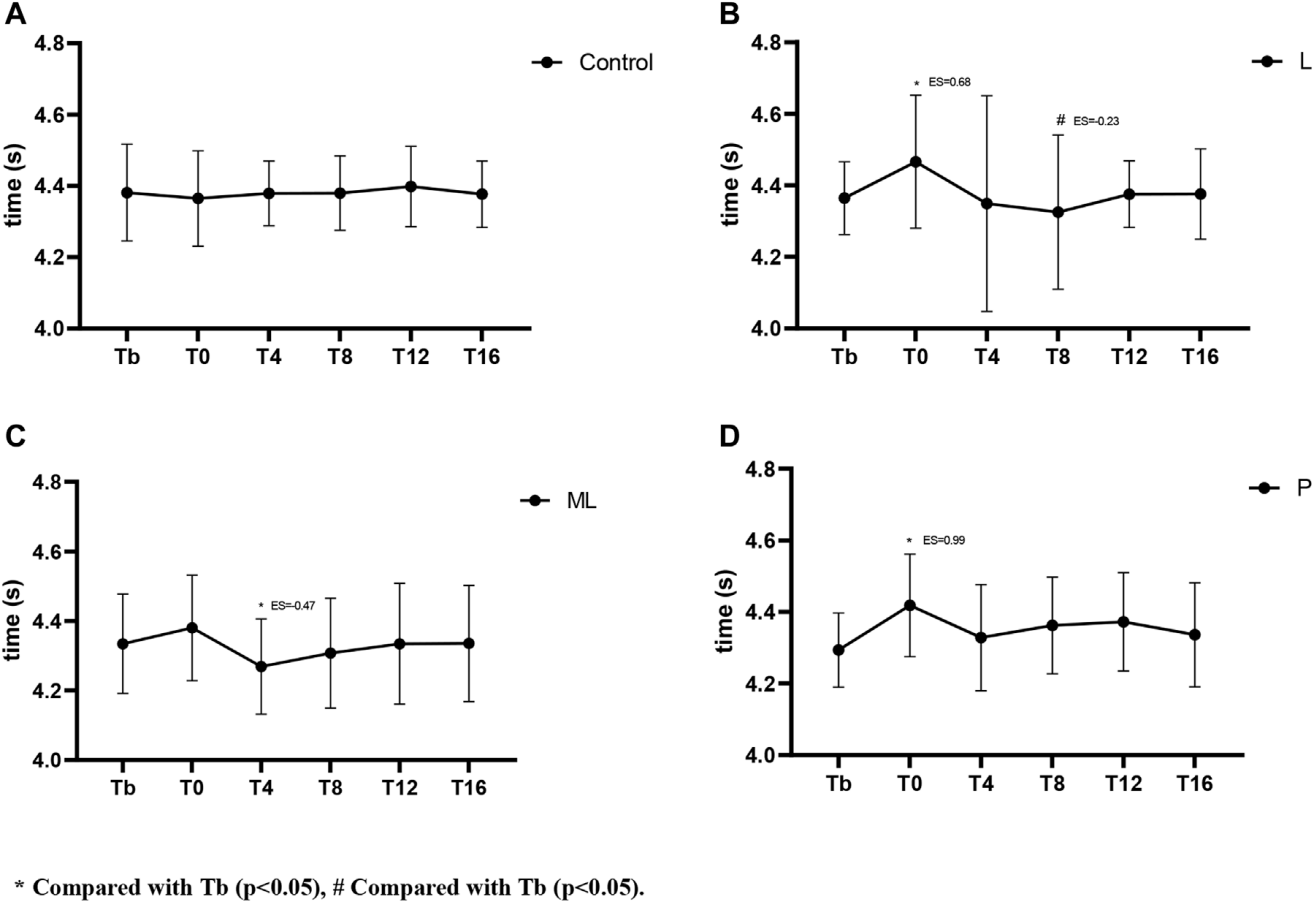
Variation of PAPE in 30 m sprint with time under different inertial loads.

The two-way repeated ANOVA showed significant differences (F(12,180) = 2.146, *p* = 0.016) of condition and time interaction on the 30 m sprint times. The simple effect showed a significant difference in sprint time when compared to the baseline Tp (*p* = 0.04) in the ML condition. Apparently, in the ML condition, the T4 showed a significant and moderate reduction in sprint time (*p* < 0.05, ES = −0.47) while no significant differences were observed in all other time conditions (*p* > 0.05). Conversely, significant moderate (*p* < 0.05, ES = 0.68) and large (*p* < 0.05, ES = 1.0) increases in sprint time were observed in the T0 time point during L and P conditions respectively (Figure 3). In addition, a small decrease in sprint time in T8 during L (*p* < 0.05, ES = −0.23) was observed. When compared with the control condition, both the L condition (*p* = 0.007) and P condition (*p* = 0.001) showed a significant increase at T0 (Figure 6A).

**FIGURE 4:**
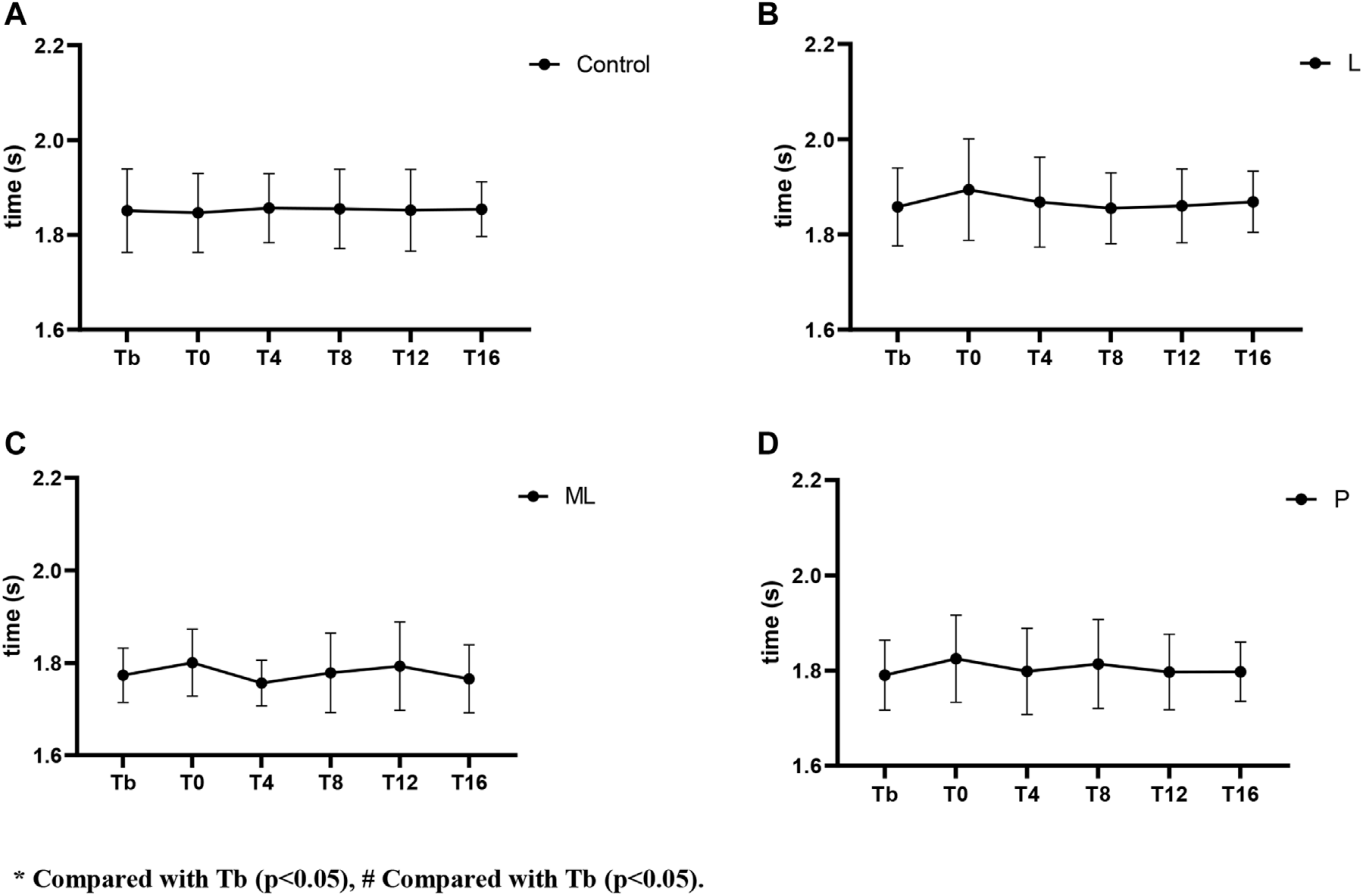
Variation of PAPE from 0-10 m in 30 m sprint with time under different inertial loads.

**FIGURE 5:**
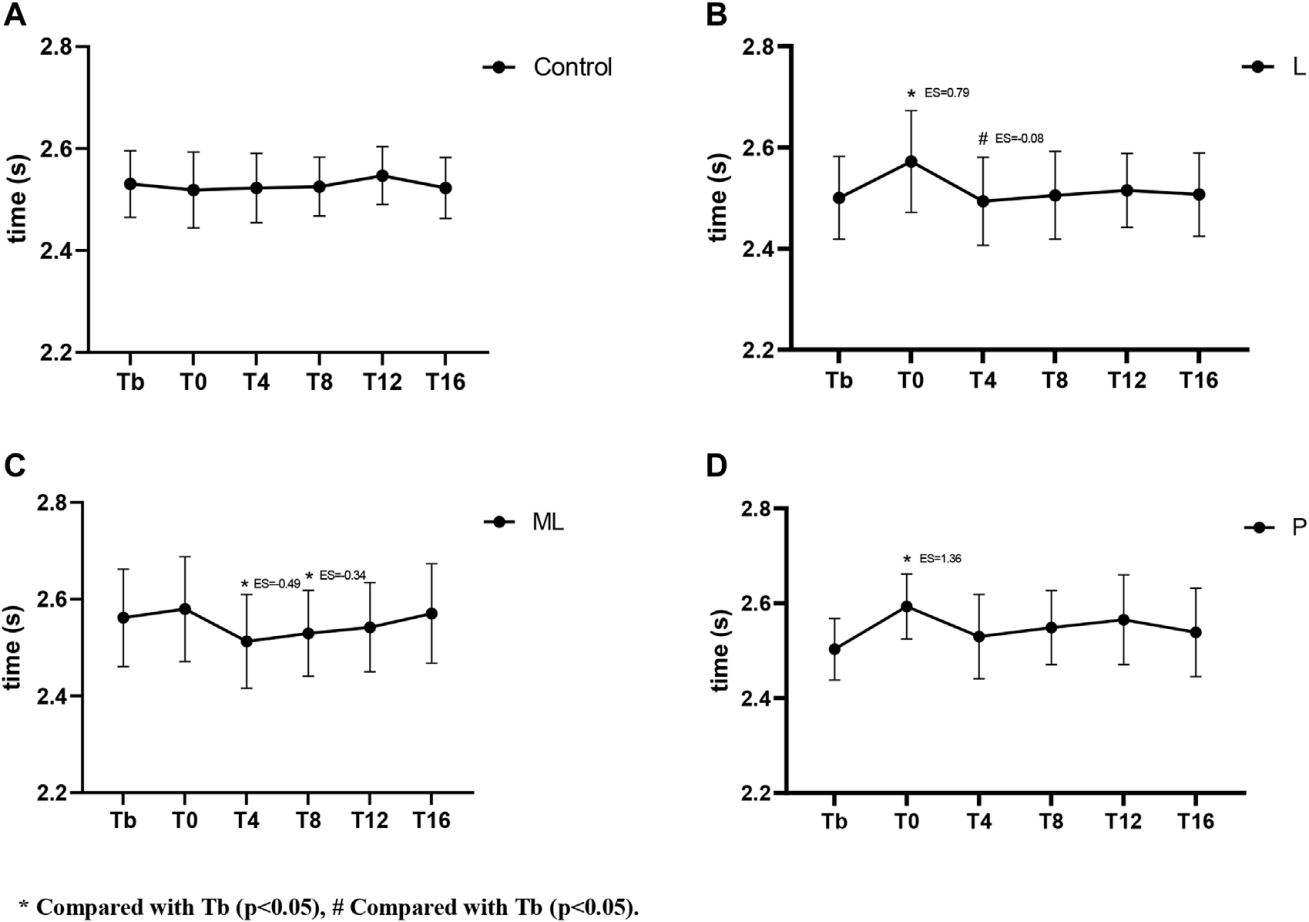
Variation of PAPE from 10-30 m in 30 m sprint with time under different inertial loads.

**FIGURE 6:**
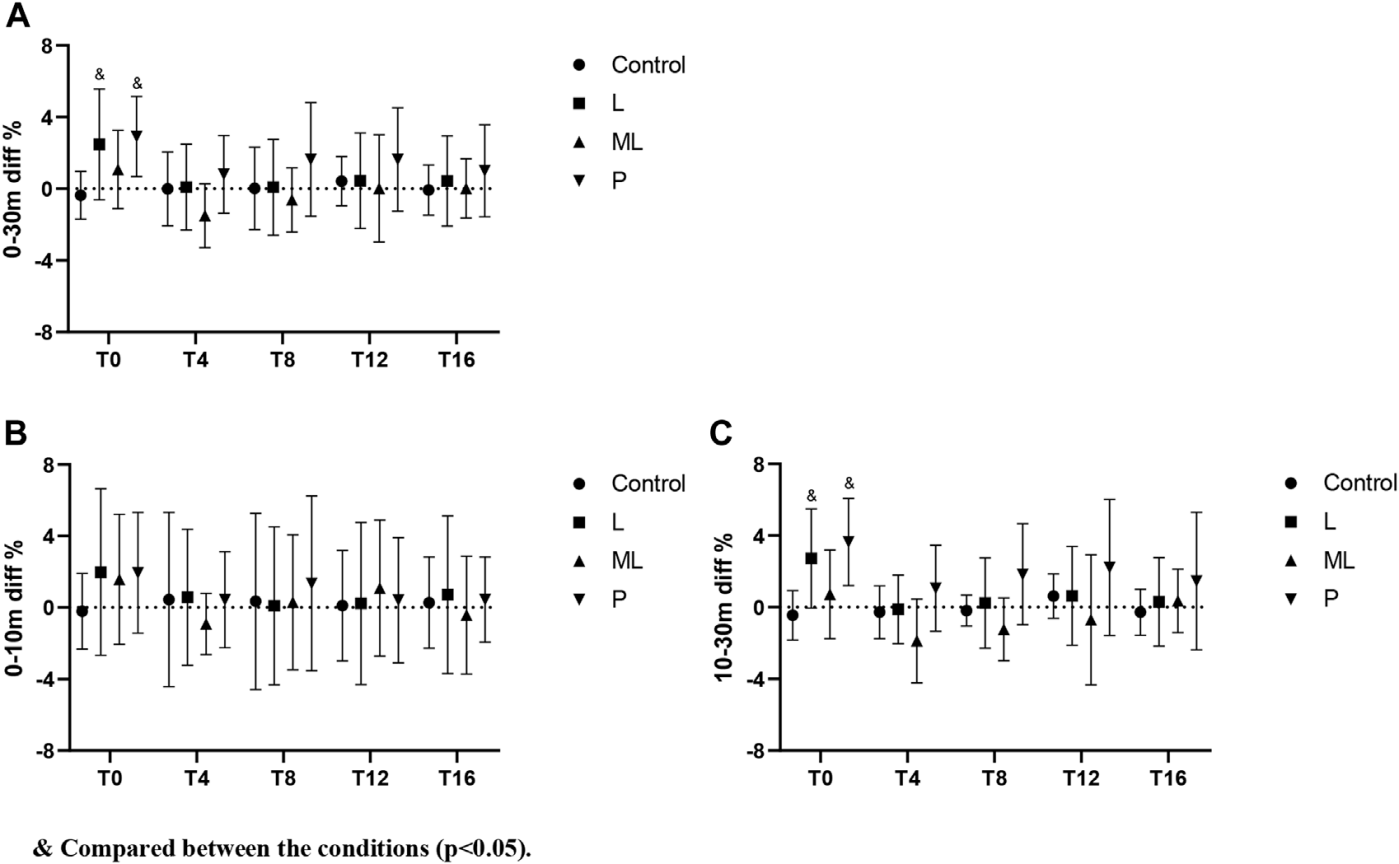
Percentage change of PAPE with time under different inertial loads.

The results of the 30 m sprint split time showed no significant difference in 0–10 m either in between or within the condition comparisons (Figures 4, 6B). The effect of condition × time interaction on PAPE during 10-3 m split time showed a significant difference (F(5,92) = 9.654, *p* < 0.001) while the simple effects showed a significant decrease in the ML condition at T4 (*p* < 0.05, ES = −0.49) and T8 (*p* < 0.05, ES = −0.34) (Figures 5, 6C).

## 4 Discussion

The current study investigated the PAPE of FT on lower limb explosive power performance. Regarding the vertical explosive power (i.e., CMJ), our results showed a general trend that the magnitude of peak PAPE was positively related to the size of inertial loads applied (P > ML > L). Except for the insignificant result in the low load (L) condition, peak PAPE appeared at T4 and mostly subsided at T12 in both ML and P conditions. When the large inertial load (*p* = 0.122 kg·m^2^) was applied, the PAPE was induced immediately (T0). Therefore, our optimum time window for yielding PAPE in the CMJ task was 0–12 min post-intervention. Conversely, The PAPE of horizontal explosive power (i.e. 30 m sprint) showed a distinct characteristic. The 30 m sprint time was slower than the baseline immediately (T0) after the FT using P and L inertial loads. Only the ML condition showed some beneficial effects at T4 (ES = −0.47) without any significant detrimental effects at other time points. When a light inertial load was used (L), only a small beneficial effect (ES = −0.23) was observed at T8. All the observable PAPEs took place between a 10–30 m split, while no significant PAPE was observed between a 0–10 m split.

Several mechanisms for enhancing lower limb performance acutely using heavy resistance exercise, weightlifting, and FT have been described in the literature (Beato et al., 2019; Beato et al., 2020; Beato et al., 2021a). The most commonly cited physiological factors underpinning the performance enhancement effect included the increased calcium sensitivity of the actin-myosin interaction and better myosin light chain (MLC) phosphorylation. Traditionally termed “post-activation potentiation” (PAP), these cascades of events have been proposed to increase the rate of cross-bridge formation and the transient enhancement of muscles’ contractile capacities, force output, power development, and the rate of force development (Tillin and Bishop, 2009; Boullosa et al., 2018). However, PAP is a muscle-memory mechanism and the effect typically lasts <30s (Blazevich and Babault, 2019; Boullosa et al., 2020), which may not fully explain the performance enhancement effect observed in our study. Alternatively, it is known that high-load contractions increase significantly muscle fiber temperature, and that speed contractions (i.e., RFD) are highly dependent on the muscle temperature because of the ergogenic effect obtained in the muscle mechanics with the increase of the temperature e.g., reduction of the muscle viscosity, an increase of the nerve conduction velocity, and an increase of the water content favoring cross-bridge attachment (Blazevich and Babault, 2019). Furthermore, nerve impulses to the muscle and the H-reflex were thought to be enhanced via the proper preload of muscles from a central perspective. All these proposed mechanisms may collectively explain the observable improvements in the explosive performance of lower limb tasks in the current study.

To achieve the best transformation of PAPE and lower limb explosive performance, the existing literature recommended performing the FT 4–12 min before the formal training tasks or competition (Tillin and Bishop, 2009; Dobbs et al., 2019). One particular study also highlighted the use of FT using medium (0.029 kg·m^2^) and high inertial load (0.061 kg·m^2^) to enhance the CMJ performance (8.5%–11.3%) (Beato et al., 2021a). Interestingly, our study only partially echoed their findings. In the study of Beato et al. (2021b), the CMJ performance dropped almost immediately (30 s post-intervention) in both their medium and high load conditions whereas no immediate detrimental effect on CMJ performance was observed in the current one. Furthermore, our CMJ even improved immediately (T0) when the very high inertial load (0.122 kg·m^2^) was applied. The authors of previous studies believed that the dominant fatigue effect had masked the observable PAPE and therefore, a sufficient post-exercise recovery interval is required to dissipate the accumulated fatigue in the FT training. However, it is worth noting that most of the previous studies used a shorter (2 min) inter-set rest during FT exercises, whereas the use of a relatively longer inter-set rest (3 min) in our study may explain the discrepancy of immediate PAPEs. Therefore, it is speculated that the sufficiently long inter-set recovery period (i.e. 3 min or above) or even adopting the cluster set configurations (e.g., intra-set rest) is the key to efficiently dissipating the fatigue and offsetting the residual dominant fatigue immediately after the FT implementation. Further studies in this regard are warranted to fully understand the rest configuration, FT-induced fatigue, and the immediate PAPE.

In addition, the aforementioned study by Beato et al. (Beato et al., 2021a) also supposed a superior effect of using a high inertial load over the medium one as theoretically, the eccentric overloaded method could better recruit higher order motor units, and induce greater postsynaptic potential and H-wave. Although their findings did not show a significant difference between high and medium load, it was proposed that a longer recovery interval (>6 min) may be required to support this supposition. In this regard, our study showed a larger magnitude of PAPE on CMJ tasks when the higher inertial load was used (P: 0.122 kg·m^2^ > ML: 0.057 kg·m^2^ > L: 0.041 kg·m^2^) while the peak PAPE appeared within 6 min timeframe (i.e. 4 min in our study). It is noteworthy that our middle load (ML: 0.057 kg·m^2^) approximated their high load (0.061 kg·m^2^) while our largest one in the P condition was twice their high load. Despite the different findings in these two studies, our results supported their supposition. Considering that a 3-min inter-set rest is barely sufficient to eliminate all the cumulated fatigue and to maintain the repetition sustainability or the lifting performance in the continuous straight-set configuration (Ho et al., 2021), the shorter inter-set rest (2 min) adopted in the previous studies might therefore hinder the expression of the potential PAPEs in using high inertial load (Beato et al., 2019; Beato et al., 2020; Beato et al., 2021a). Therefore, we recommend that practitioners may use multiple sets of ML (0.057 kg·m^2^) and P (0.122 kg·m^2^) inertial loads of FT interspersed with 3-min inter-set rest meanwhile giving a 4-min post-FT recovery period to peak the PAPE before vertical jump-related activities or training. For shorter inter-set rest (e.g., 2 min) during EOL training, with the reference from previous findings, the inertial load should be adjusted to light and moderate (≤0.061 kg·m^2^) while a 6-min post-FT recovery window is needed to achieve the peak PAPE. When considering the optimal balance between FT loading stimulation and induced fatigue, a recent study has shown a significantly higher concentric and eccentric peak power output in FT-assisted squat over the classic FT squat with an unassisted concentric phase (Wren et al., 2023). Therefore, it is an interesting question if FT-assisted squat can potentially yield a higher magnitude and longer duration of PAPE on jumping and sprinting tasks with the same or even lower FT volume.

Besides the interaction between loading and recovery period, the FT volume and intensity may also play a role in PAPE. A recent study has shown that multiset (at least two) FT half-squat exercises (light inertia with 0.029 kg·m^2^) were required to elicit significant PAPE on jumping performance after 3 or 6 min (de Keijzer et al., 2020). Interestingly (Maroto-Izquierdo et al., 2020) have successfully demonstrated a single set of high-intensity FT (0.083 kg·m^2^) that led to a peak PAPE with a small effect on CMJ enhancement after 12 min and meanwhile, a clear increase of peak concentric velocity was still observable after 20 min. It seems that proper dosage of FT exercises is essential to optimize the PAPE (maximize the magnitude and duration) of jumping performance. Given that a lower volume of FT might potentially decrease the magnitude and duration of fatigue while a sufficiently high intensity of stimulation is needed for maximizing the neuromuscular response, it is speculated that a lower volume of high-intensity FT (i.e., 2 sets of our P condition) may potentially achieve comparable jumping or sprinting enhancement meanwhile lengthen the duration of observable PAPE (e.g., only diminished after 16 or 20 min in CMJ or sprinting tasks). Future studies regarding the different combinations of intensity and volume to identify the optimal FT dosage on PAPE are indicated.

Regarding the horizontal explosive tasks, since PAPE using FT has the property of task-specific adaptations (Beato and Dello Iacono, 2020), the PAPE should only be maximized when the PAPE drill and the performing activities share similar biomechanical characteristics (e.g., vector force and joint movements) (Beato et al., 2020). Therefore, even though similar muscles were recruited in squat and sprint tasks, the FT drill using half squat might only yield sub-optimum PAPE on sprinting performance. In support of previous studies, our findings showed a similar trend in the expression of fatigue and PAPEs after FT exercises that the sprint performance decreased (longer sprint time) immediately (T0) in all conditions (Figures 3–5). From the magnitude perspective, modest beneficial PAPEs on 10–30 m sprint tasks were observed (only in ML condition at T4 with ES = −0.49), while both L and P yielded no or trivial effects in most sprint situations. It seems possible that the low inertial load in L did not provide sufficient stimulation, while the high load in P may induce too much fatigue and undermine the PAPE during the subsequent recovery period. Therefore, FT as a preload modality may induce the PAPE to different extents according to the nature of the subsequent training or performing activities, while the potential PAPE benefits can be retained in the recovery period depending on 1) the total amount of PAPE and fatigue produced during training and; 2) the residual PAPE and fatigue during a range of recovery period. Besides, the motor pattern interference effect, or called “perseveration” (Giannouli, 2013) that the initial task perseveres and leads to the perceived loss of coordination of the second similar task may have played an important role in hindering the post-FT PAPE. Apart from the biomechanical differences between squat, CMJ, and sprint, the long ground contact time of CMJ (>0.25 s) can be regarded as a slow stretch-shortening cycle (SSC) while the 30 m sprint is dominated by the fast SSC activities. Thus, this may further explain that the relatively slow FT squat without any SSC involvement did not maximize the PAPE in transferring to the sprinting activities. In this regard, the previous study showed a significant improvement in both the 10 and 20 m sprint performance (2.3%–2.6%) in the window between 4 and 8 min after weighted (10% of body mass) plyometric PAPE drills (Turner et al., 2015). Further studies to compare the actual PAPE differences on sprint tasks after FT, plyometric exercises, and the combined methods are required to provide more conclusive practical guidelines in this area.

This study has several strengths, including using a crossover randomized controlled trial that can eliminate the issues of between-subjects differences. At the same time, most previous studies adopted 2-min inter-set rest, which might have more prominent cumulated fatigue. In return, this might potentially mask the PAPE expression. The longer inter-set rest (3 min) used in our study seemed to be more capable of unmasking the PAPE potentials in wider perspectives and conditions.

Despite these strengths, major limitations of the present study included that only the vertical FT (parallel squat) was adopted. The optimum FT exercise format to maximize CMJ and sprint performance should be further determined by comparing the PAPEs with other movements (e.g., unilateral-based such as lunge and horizontal-based such as hip thrust). Meanwhile, our findings have included T0 (or within 15s after FT) and both PAP and PAPE probably co-existed in around 30 s (Blazevich and Babault, 2019). The current experimental design was not able to differentiate and explain the determinant effect (PAP vs. PAPE) of any observable performance change of this condition. In the future, muscle activity monitoring such as electromyography can also be used to evaluate the degree of motor unit recruitment and the biomechanical similarity. Moreover, given the fact that performance enhancements would not depend only on the fiber type II stimulation, another future study perspective is to test if light loads could trigger the same effects as heavy loads but with higher repetitions. Finally, further investigations can explore the use of assisted flywheel squats to determine if such an approach could be suitable for inducing PAPE.

## 5 Conclusion

In conclusion, this study shows that the multiple sets of FT method as the preloaded activity can acutely enhance the CMJ and 30 m sprint performance in trained individuals. With 3-min inter-set rest given during the preloaded activity, both P and ML inertial loads could produce the peak PAPE and CMJ performance at 4 min and the benefits gradually subsided after 12 min, whereas the 30 m sprint (especially the 10–30 m split) was enhanced via PAPE using ML inertial load at 4–8 min time points. Future research is encouraged to further explore the optimum FT exercise format to maximize the PAPEs on various exercise tasks.

## Data Availability

The original contributions presented in the study are included in the article/Supplementary Material, further inquiries can be directed to the corresponding authors.
